# Role of oceanography in shaping the genetic structure in the North Pacific hake *Merluccius productus*

**DOI:** 10.1371/journal.pone.0194646

**Published:** 2018-03-26

**Authors:** Francisco Javier García-De León, Carolina Galván-Tirado, Laura Sánchez Velasco, Claudia A. Silva-Segundo, Rafael Hernández-Guzmán, Irene de los Angeles Barriga-Sosa, Píndaro Díaz Jaimes, Michael Canino, Pedro Cruz-Hernández

**Affiliations:** 1 Laboratorio de Genética para la Conservación, Centro de Investigaciones Biológicas del Noroeste, La Paz, Baja California Sur, México; 2 Instituto Politécnico Nacional, Centro Interdisciplinario de Ciencias Marinas, Departamento de Plancton y Ecología Marina, La Paz, Baja California Sur, México; 3 CONACYT–Instituto de Investigaciones sobre los Recursos Naturales, Universidad Michoacana de San Nicolás de Hidalgo, Av. San Juanito Itzícuaro s/n, Morelia, Michoacán, México; 4 Laboratorio de Genética y Biología Molecular, Planta Experimental de Producción Acuícola, Universidad Autónoma Metropolitana Unidad Iztapalapa, Av. San Rafael Atlixco 186. Col. Vicentina, Del. Iztapalapa, Cd. de México, México; 5 Instituto de Ciencias del Mar y Limnología, Universidad Nacional Autónoma de México, Ciudad Universitaria, Cd. de México, México; 6 Alaska Fisheries Science Center, Seattle, WA, United States of America; 7 Laboratorio de Genética Acuícola, Centro de Investigaciones Biológicas del Noroeste, La Paz, Baja California Sur, México; National Cheng Kung University, TAIWAN

## Abstract

Determining the relative influence of biotic and abiotic factors on genetic connectivity among populations remains a major challenge in evolutionary biology and in the management and conservation of species. North Pacific hake (*Merluccius productus*) inhabits upwelling regions in the California Current ecosystem from the Gulf of California to the Gulf of Alaska. In this study, we examined mitochondrial DNA (mtDNA) and microsatellite variation to estimate levels of genetic differentiation of *M*. *productus* in relation to the role of oceanographic features as potential barriers to gene flow. Samples were obtained from nine sites spanning a large part of the geographic range of the species, from Puget Sound, Washington to Costa Rica. The microsatellite results revealed three genetically discrete populations: one spanning the eastern Pacific coast, and two apparently resident populations circumscribed to the Puget Sound and the northern Gulf of California (F_ST_ = 0.032, p = 0.036). Cytochrome b sequence data indicated that isolation between the Puget Sound and northern Gulf of California populations from the coastal Pacific were recent phenomena (18.5 kyr for Puget Sound and 40 kyr for the northern Gulf of California). Oceanographic data obtained from the Gulf of California support the hypothesis that permanent fronts within the region, and strong gradients at the entrance to the Gulf of California act as barriers to gene flow. A seascape genetics approach found significant genetic–environment associations, where the daytime sea surface temperature and chlorophyll concentrations were the best predictive variables for the observed genetic differentiation. Considering the potential causes of genetic isolation among the three populations, e.g. spawning areas in different latitudes associated with upwelling processes, oceanographic barriers, asymmetric migration and specialized diet, oceanographic barriers appear to be a likely mechanism restricting gene flow.

## Introduction

Resolving subtle genetic structure in marine species where barriers to gene flow are not apparent is a central issue both for evolutionary biology and in the management and conservation of species [1–4]. A large number of studies on the basic mechanisms of genetic connectivity in marine fish (*e*.*g*., [5–11]) emphasize that biotic (time and place of spawning, pelagic larva duration, homing, etc.) and/or abiotic (geographic distance, currents, oceanic fronts, differences in salinity, temperature, etc.) factors can shape subtle genetic structures. Vicariance is usually invoked to explain genetic discontinuities across contemporary, contiguous geographic ranges [12–14]. However, the genetic marker class, frequency and spatial distribution of sampling, and conceptual considerations (*e*.*g*., interpretations of the relative effects of genetic drift and high dispersal potential of species with large effective population sizes) have sometimes yielded contradictory results [10,15] Seascape genetics employs a set of conceptual and methodological tools that attempt to link oceanographic features with population genetics to determine how temporal and spatial factors influence distributions of genetic variation [16,17].

North Pacific hake, also known as Pacific whiting, has a temperate demersal distribution largely restricted to continental shelves and slopes from 45 to 500 m deep [18]. A recent review of morphological and mitochondrial DNA (mtDNA) data by Silva-Segundo et al. [19] determined that *M*. *productus* represents a single species distributed from Washington State to Costa Rica. Four populations or stocks have been proposed in this region based upon morphology, behavior and life history traits [18–23]. One highly migratory stock inhabits the continental shelf from Vancouver, British Columbia to Magdalena Bay, Mexico [19,20]. Two resident stocks occur in mostly isolated waters of the Salish Sea (Strait of Georgia and Puget Sound) and another “dwarf” stock is confined to the southwest coast of southern Baja California Peninsula [18,20,21]. The genetic distinctness of these stocks was confirmed except for dwarf hake [22]. Recently, another population was recognized inside the Northern Gulf of California [23]. However, despite a recent review of the taxonomic status of *M*. *productus* [19], there is still controversy over whether to recognize the Northern Gulf of California hake population as a distinct species, *M*. *hernandezi* [24–26].

The highly migratory behavior of hake is apparently reflected in broad-scale genetic panmixia or subtle genetic differentiation (*i*.*e*. weak isolation-by-distance) reported for some Atlantic and Pacific hake species along coasts [11,22,23,27–29]. Stronger genetic differentiation for resident stocks inhabiting partially-isolated geographic areas has also been reported in some hake species; i.e. between Atlantic and Mediterranean populations of *M*. *merluccius* [10,14], between the Gulfs of St. George and St. Matthias and the Atlantic ocean in *M*. *hubbsi* [28,30], between the Gulf of Maine and Atlantic ocean in *M*. *bilinearis* [28], and between the Puget Sound, Gulf of California and the Pacific Ocean in *M*. *productus* [22,23]. These results would appear contradictory given the assumed high dispersal capacity in these species. However, phylogeographic breaks between populations of different fish taxa within the Gulf of California and the contiguous Pacific Ocean have been documented, primarily from mtDNA analyses, suggesting that the entrance to the Gulf of California has acted as an historical barrier to gene flow for many species with diverse life histories [12,31] but not for others [12,32]. It has been theorized that population isolation is mediated by mesoscale oceanographic processes in the entrance to and adjacent waters of the Gulf of California acting as barriers to larval dispersal [33,34]. A similar phenomenon occurs in Puget Sound, where large-scale reflux via mixing sills and small-scale circulation patterns (e.g. nearshore eddies) may affect larval dispersal and limit gene flow among populations of several species [22,35–37]. To date, there has been no attempt to examine oceanographic factors influencing genetic structure in North Pacific hake. In this study, we asked whether genetically distinct populations exist in regions of environmental heterogeneity. We were also interested in estimating the times since divergence of the putative stocks, and whether some have remained demographically stable. We reanalyzed mtDNA and microsatellite data published in Silvia-Segundo et al. [19] and Iwamoto et al. [23], respectively, using two sets of oceanographic data: one obtained from research cruises, which characterized frontal zones and hydrographic gradients in the Gulf of California, plus physical and biological variables obtained from a web database (Ocean Color Web, https://oceancolor.gsfc.nasa.gov). A seascape genetics approach compared genetic heterogeneity between resident and coastal stocks in relation to oceanographic conditions. Finally, we used mtDNA sequence variation to estimate divergence times among these populations.

## Material and methods

The samples were not obtained in protected natural areas, national parks or private areas, so for Mexico CONAPESCA (National Commission of Aquaculture and Fishery) issued the permissions for sample collection, and for the North American hake the authorities who issued permission for sample collection were the states of Alaska, Washington, Oregon and California. The species that supporting this study species is not endangered or protected species in according to Comisión Nacional para el Conocimiento y Uso de la Biodiversidad México (CONABIO) http://www.biodiversidad.gob.mx/especies/especies_enriesgo/buscador_especies/espRiesgo.php., and The IUCN Red List of Threatened Species, http://www.iucnredlist.org/. No anesthesia protocol was followed because the specimens were captured in deep waters with trawl nets and when the fish reached surface, they were already dead, so they were dissected directly after capture.

### Databases

**Fig 1:**
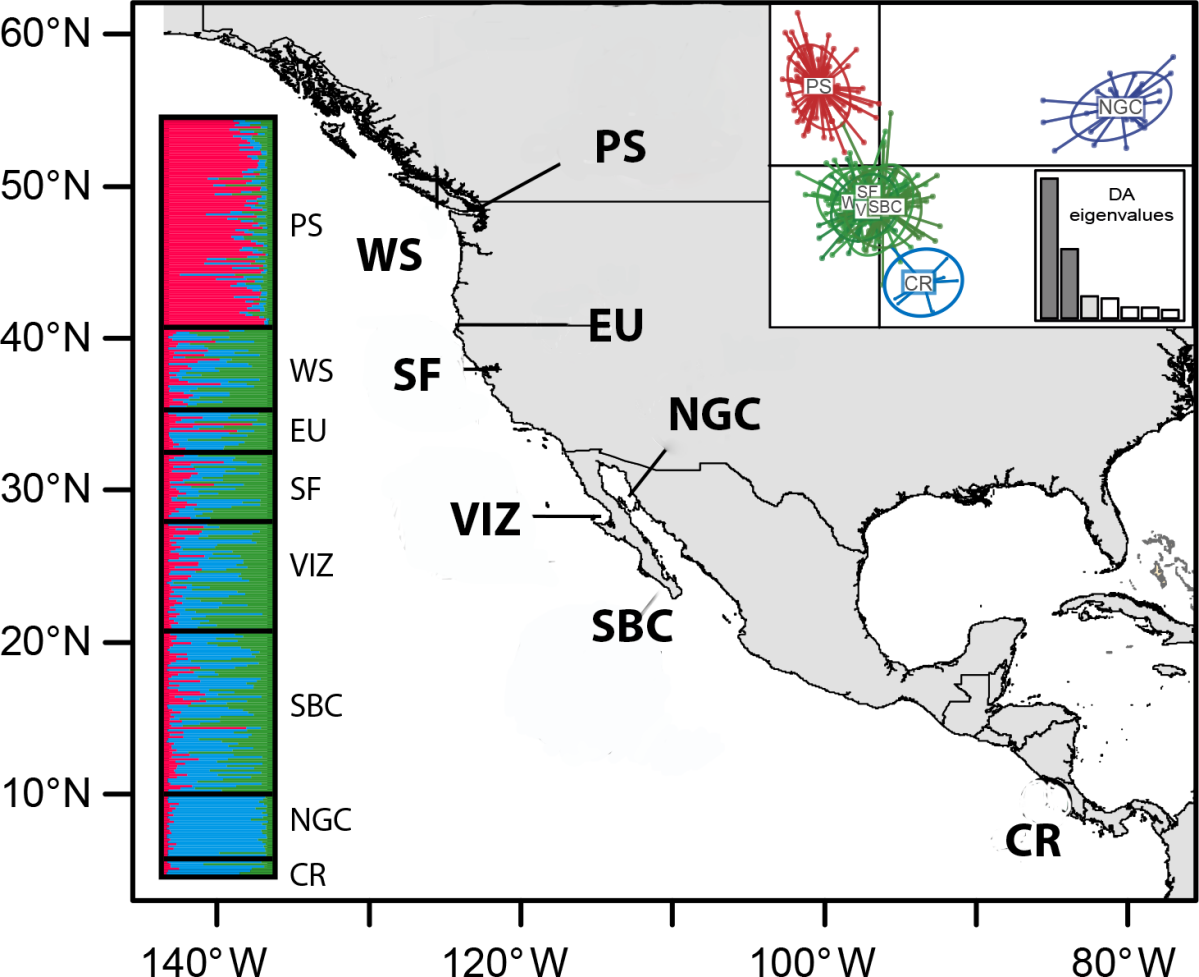
Sampling localities of *Merluccius productus* and results from microsatellite analysis. Sampling location abbreviations are as in Table 1. Left chart shows proportional assignment of individuals (horizontal bars) to genetic cluster identified by STRUCTURE (blue NGC, green PC, red PS). Right upper chart shows the Discriminant Analysis of Principal Components (DAPC).

We analyzed data obtained in previous studies [19,23] consisting of individuals from nine locations: Puget Sound (PS), Washington (WS), Oregon (OR), Eureka (EU), and San Francisco (SF) in the United States; Vizcaino (VIZ), Southern Baja California Peninsula (SBC), and the northern Gulf of California (NGC) in Mexico; and Costa Rica (CR) (Fig 1). In the previous microsatellite study [23], only some of the samples corresponding to PS and NGC were analyzed (Table 1 and Table A in S1 Table, supplementary material). We added new microsatellite data from WS, EU, SF, VIZ, SBC and CR. For details of the sampling methods and laboratory analysis see [19,23]. The microsatellite data is presented as Supplementary material (S2 Table). Descriptions of the ecosystems for the collective group of Pacific Costal stocks (PC), which include WS, OR, EU, SF, VIZ and SBC, and resident populations (PS and NGC) are given in S1 Text in Supplementary material.

**Table 1:** Indices of genetic diversity from mtDNA sequences and microsatellite loci for *Merluccius productus*.

Location	Abbreviation	Mitochondrial DNA					Microsatellites				
		N	H %	H_P_ %	h	π	N	A_R_	A_P_	Ho	He
Puget Sound	PS	14	4 7.8	3 7.0	0.396 ± 0.159	0.000 ± 0.000	93	7.60	1.28	**0.82**	**0.87**
Washington	WS	17	6 11.8	3 7.0	0.779 ± 0.074	0.001 ± 0.001	37	9.02	1.34	**0.88**	**0.93**
Oregon	OR	16	8 15.7	4 9.3	0.808 ± 0.093	0.001 ± 0.001	NA	NA	NA	NA	NA
Eureka	EU	4	2 3.9	0 0	0.5 ± 0.265	0.000 ± 0.001	19	9.17	1.41	**0.85**	**0.92**
San Francisco	SF	18	6 11.8	2 4.7	0.679 ± 0.109	0.001 ± 0.001	31	8.98	1.31	**0.87**	**0.92**
Vizcaino	VIZ	10	8 80.0	3 30.0	0.956 ± 0.059	0.003 ± 0.002	49	9.21	1.44	**0.86**	**0.94**
Southern of Baja California	SBC	14	9 15.7	5 9.3	0.879 ± 0.079	0.002 ± 0.001	73	9.46	1.77	**0.84**	**0.94**
Northern Gulf of California	NGC	23	20 39.2	20 46.5	0.984 ± 0.019	0.003 ± 0.002	29	8.90	2.43	**0.89**	**0.91**
Costa Rica	CR	8	6 11.8	3 7.0	0.893 ± 0.111	0.002 ± 0.001	7	9.70	2.39	0.89	0.86
Total		124	51	43	0.858 ± 0.029	0.003 ± 0.002	338	9.02	1.67	0.85	0.94

Numbers of individuals (N), haplotypes (H) and private haplotypes (HP; also expressed as a percentage of the total number of samples), haplotype diversity (h), nucleotide diversity (π), average allelic richness (A_R_), private allelic richness (A_P_), observed (Ho) and expected (He) heterozygosities for Pacific hake. Significant values for heterozygote deficits in bold (P <0.001).

### Statistical analysis

#### Genetic diversity

Mitochondrial DNA sequences were edited and aligned using the ClustalW program implemented in MEGA 6 [38]. Best-fitting substitution models for each mtDNA gene alignment were determined by jModelTest 2 according to Akaike and Bayesian information criteria [39]. Mesquite v3.31 [40] was used to produce a concatenated alignment for analyses. Molecular diversity indices, such as haplotype (*h*) and nucleotide (*π*) diversity, were estimated using Arlequin 3.5 [41].

Microsatellite data were examined for scoring errors and null alleles using FreeNA [42]. Deviations from Hardy–Weinberg equilibrium (HWE) and linkage disequilibrium were determined for each locus and location with GENEPOP 4.0 [43]. Estimates of genetic diversity, such as observed (Ho) and expected (He) heterozygosities were calculated with GENALEX [44]. Rarefaction was used to estimate standard private allelic richness (AP) and allelic richness (*A*_R_) for each locus and location for random subsamples of nineteen genes, the minimum sample size (CR) in the study with HP-RARE v1.0 [45]. This method compensates for uneven sample sizes and accommodates hierarchical sampling designs; see Kalinowski [46] for more details. A Student's t-test was done comparing samples with the smallest and largest averages for each parameter of genetic diversity in the Excel platform.

We used the F_ST_ outlier approach [47,48] implemented in LOSITAN v1.0.0 [49] to detect putative loci responding to selection. This method examines the distribution of F_ST_ vs. H*e* to identify loci with unusually high or low F_ST_ values [49]. For this analysis, we used 100,000 simulations, a “neutral” mean F_ST_ (potentially non-neutral loci are removed prior calculating the initial mean), 99.5%confidence intervals and a false discovery rate (FDR) of 0.05 assuming infinite alleles and stepwise mutation models.

#### Genetic differentiation

Simulations in POWSIM v 4.0 [50] were used to evaluate the power of the microsatellite data to detect genetic differentiation using both Fisher’s exact test and traditional Chi-square approaches. Three different levels of genetic differentiation were tested: F_ST_ = 0.05 (N*e* = 7000, generations since divergence (t) = 750), F_ST_ = 0.01 (N*e* = 7000, t = 150) and F_ST_ = 0.001 (N*e* = 7000, t = 20). All simulations were performed for eight populations (sample sites). In all cases, 1,000 replicates were run and the percentage of significant outcomes (α = 0.05) for a range of predefined F_ST_ values was interpreted as the power of the tests for detecting that level of differentiation. Pairwise sample F_ST_ values for both mtDNA (ϕ_*ST*_) and microsatellites (F_*ST*_) were estimated with Arlequin 3.5 [41] using 10,000 data permutations. A sequential Bonferroni correction was applied for multiple tests [51].

Mitochondrial DNA and microsatellites data were analyzed for hierarchical population structure with an analysis of molecular variance (AMOVA) using four grouping strategies: (A) assuming all localities as different populations; (B) pooling coastal localities from PS to SBC, with NGC and CR as different populations; (C) pooling all coastal samples and assuming PS and NGC as two different populations; and (D) pooling coastal samples with PS, NGC and CR considered as separate populations. An isolation-by-distance (IBD) analysis was performed on the microsatellite data. Shoreline distances (km) between sampled populations were estimated using Google Earth 4.3 and plotted against pairwise genetic distance [52]. IBD regressions were performed online using the IBD web service (IBDWS, http://phage.sdsu.edu/~jensen/) [53] with 10,000 data randomizations. The extent of population differentiation among samples using microsatellites was visualized with discriminant analysis of principal components (DAPC) with the adegenet application [54] implemented in R (R Development Core Team, 2013). Bayesian analysis was used to estimate the number of putative populations from the sample data using STRUCTURE 2.3.4 [55]. To estimate the number of populations (K), 15 independent simulations for K = 1–7 were run using 1,000,000 data iterations after discarding the first 250,000 as burn-in. Analyses were performed with the admixture model of population structure and independent allele frequencies among populations. We used the LOCPRIOR option, with the most likely K determined using Evanno’s ΔK [56,57]. Graphical representation of STRUCTURE results were performed using CLUMPAK (http://clumpak.tau.ac.il/index.html).

Migration rates among populations were estimated using microsatellite data with Migrate-n 3.6.5 [58]. We conducted searches using one long and four short MCMC chains with a sampled genealogy of 1,000,000 set at increments of 200, after discarding the first 200,000 trees as burn-in. Migration rates were estimated between the pooled coastal stock and NGC and PS samples. Potential genetic barriers associated with geographic locations for both mtDNA and microsatellite data sets were visualized with Monmonier’s maximum-difference algorithm implemented in Barrier 2.2 [59].

#### Historical demography

A haplotype genealogy for mtDNA data was obtained using Haploview v4.2 [60]. Inferences for patterns of historical demography were inferred from the distribution of nucleotide mismatches using Arlequin 3.5 [41]. A mismatch distribution was constructed for each sample group to test the goodness of fit to a unimodal distribution [61] of exponential population growth [62] using Harpending’s raggedness index (*r*) and the simulated sums of squared deviation (*SSD*). Tajima’s *D* [63], and Fu’s *F*s [64] neutrality tests were calculated by population using 10,000 data permutations.

Divergence times were estimated using BEAST v1.8.0 [65]. We used CYTB sequences from five hake species obtained from Genbank to construct the phylogenetic tree and estimate divergence times (Table B in S1 Table, supplementary material). The HKY+I+Γ nucleotide substitution model and estimated lognormal relaxed clock were used with a coalescent of constant size, as recommended for analyses of closely related species or populations [65]. Two independent replicates, each with 250,000,000 generations were simulated, with sampling every 25,000 generations. The stationarity of the MCMC chains and convergence of the two runs was monitored by Tracer v. 1.5 [66] to determinate whether the effective sample size for all parameters exceeded 200 as recommended by the manual. Files from the two runs were combined using LogCombiner v1.8.0 [65] and a consensus tree with nodal heights and 95% confidence time intervals was generated with TreeAnnotator v. 1.8.0 [65] after discarding the first 5,000 trees as burn-in. A consensus tree was drawn using FigTree v1.4.1 [65]. We used a calibration point of 15 thousand years (kyr) suggested by Iwamoto et al. [22] as the divergence time between PS and the coastal Pacific hake population, and 4,1 myr between European and American hake clades [67], and rooted the tree with a conservative estimate for closure of the Panamanian Isthmus of 3,5 myr [68,69].

#### Satellite and hydrographic data

**Fig 2:**
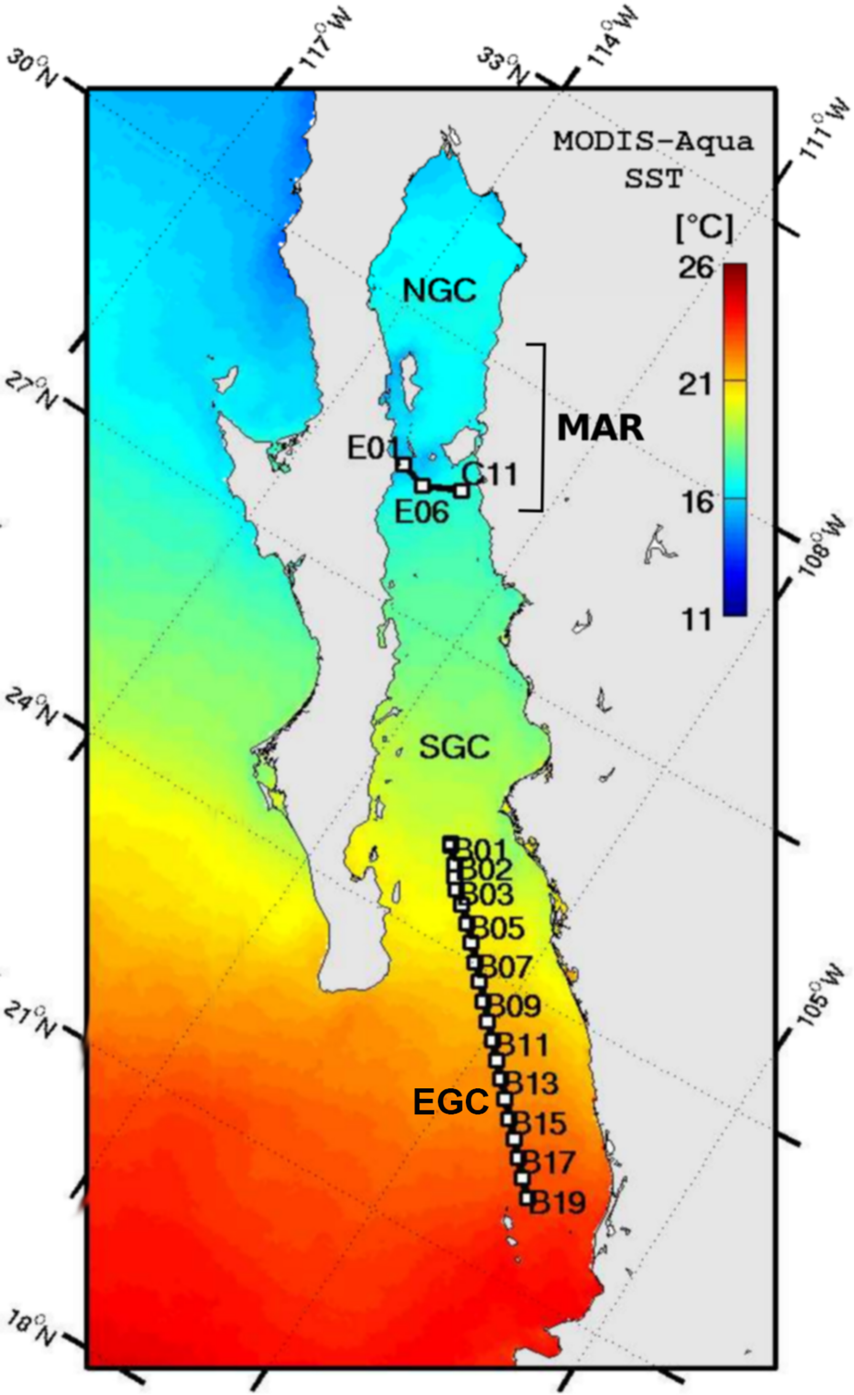
Satellite and hydrographic data. Climatology of February of the Gulf of California (2002–2010) from the MODIS-Aqua satellites: sea surface temperature (SST) °C. Midriff archipelago region (MAR), northern Gulf of California (NGC), south of the Gulf of California (SGC), and entrance of Gulf of California (EGC).

The presence of fronts and hydrographic gradients in the Gulf of California was determined during winter, the main spawning season of hake in that region [70]. Mean February sea surface temperature (SST) from 2000–2010, obtained from the Aqua-MODIS satellites (4 km × 4 km resolution), were analyzed along with hydrographic data from two oceanographic cruises (GOLCA0702: 19 February -1 March 2007) covering 90 stations in the Midriff archipelago region (MAR) and GOLCA1002:14–27 February, 2010 from 68 stations in the entrance of the Gulf). Cruise transects crossed two features of interest in this study: (1) the frontal zone south of the MAR, and (2) strong hydrographic gradients at the entrance to the Gulf of California (Fig 2). Vertical temperature and salinity profiles were obtained at each station across the frontal zone with a calibrated CTD (model 911plus, Sea-Bird Electronics, Bellevue, WA). Sampling methodology and processing of physical data are described in Godínez et al. [71].

#### Seascape genetic analyses

In order to relate patterns of genetic variation with environmental heterogeneity, a seascape genetics approach was developed following Selkoe et al. [72] and Henriques et al. [11]. A genetic matrix of ancestral coefficients was constructed with STRUCTURE 2.3.4, using an ancestral coefficient (q) of 0.75. Individuals with a q < 0.75 were excluded from the analyses, resulting in a final matrix of 130 individuals.

Geographic coordinates of each sampling site (Table A in S1 Table, supplementary material), excepting CR (due to low sample size) were used to construct a matrix of environmental variables. We selected complementary sites covering a mean area of 3244 km^2^ for each location (minimum 430 PS, maximum 6583 km^2^ VIZ). In total, oceanographic data from 32 sites were analyzed (three in VIZ, six in SBC, nine in NGC, five in PS, four in WS, two in EU and three in SF). Day time (SSTd) and night time (SSTn) sea surface temperatures, PAR (Photosynthetically Available Radiation), POC (Particulate Organic Carbon), water turbidity (measured as the diffusion attenuation coefficient at 490 nm), and CHL (Chlorophyll *a* concentration, mg/m^3^) were obtained from satellite data for each site. Values were extracted from 4-km spatial resolution layers (Ocean Color Web, https://oceancolor.gsfc.nasa.gov) using SeaDAS v7.4 [73] and averaged over four years (2006–2009) for four months (March—June), creating spatial windows of 3x4 pixels (12 km^2^). Averages were estimated for the time of sample collections and the seasonal occurrence of larvae in the study areas. Variables were tested for autocorrelation using Spearman non-parametric correlation tests implemented in PAST [74] and removed from subsequent analyses if significantly autocorrelated.

The role of oceanography in generating spatial genetic heterogeneity was examined in a redundancy analysis (RDA) [75] using Canoco v5 [76]. This multivariate method summarizes linear relationships between components of response (genetic) variables that are "redundant" with, i.e. "explained" by, a set of (oceanographic) variables. CHL was log-transformed and the genetic data were square-root-transformed prior to analyses [11]. We then used the BIOENV and DistLM methods [77] implemented in PRIMER v7 [78]. Both are dissimilarity-based methods that identify Euclidian distance matrices of explanatory variables for correlation with a maximum Bray-Curtis dissimilarity matrix derived from genetic data at the same sampling sites. For the DistLM analysis, variables were added stepwise to the model, using the AICc (corrected for sample size) and marginal test criteria to identify the most influential oceanographic variables after 10,000 data permutations.

## Results

### Genetic diversity

The three partial mtDNA gene fragments (CYTB, COI and 16S) were 350, 393, and 449 base pairs (bp) in length, respectively, resulting in concatenated fragment of 1192 bp. Mitochondrial DNA variation indicated a relatively high degree of global haplotype diversity (*h* = 0.858) and low nucleotide diversity (*π* = 0.003). The NGC sample contained the most haplotypes (20) and the EU sample the lowest number (2), likely due to small sample size (Table 1).

Null allele frequency estimates for 64 sample x location pairs exceeded 0.10 for two microsatellite loci at one sampling site each (0.130 for Mmer4 in WS and 0.133 for C2-1 in EU). Frequencies between 0.01 and 0.10 occurred in 53.1% of the samples, and 43.8% had nulls at < 0.01. Two microsatellite loci developed for European hake (Mmer4 and Mmer20) showed null alleles at frequencies of 0.01 and 0.130, which occurred at almost all localities (Table C in S1 Table, supplementary material). Eleven combinations deviated significantly from HWE following Bonferroni correction, nine of them due to heterozygote deficiencies (see Table D in S1 Table, supplementary material). Loci Mmer4 and Mmer20 each had similar deviations in four locations, although both showed departures from HWE only in VIZ and SBC samples. No significant linkage disequilibrium was detected in any pairwise locus x location comparisons or globally after Bonferroni correction (Table E in S1 Table, supplementary material). The two heterologous loci (Mmer4 and Mmer20) did generate significantly more HWE disequilibrium than other loci isolated from *M*. *productus*. We performed F_ST_ tests both with and without Mmer4 and Mmer20, and concluded that null allele frequencies of < 25% have minimal impact on estimates of genetic structure, and therefore decided to use information from all eight loci (see S2 Table in supplementary material).

Samples from each location had comparable and moderately high genetic diversity, with many alleles at each locus (mean *A*_*R*_ = 12.27) and moderately high observed (*Ho* = 0.85) and expected heterozygosities (*He* = 0.94). The lowest and highest mean *Ho* values (0.82 and 0.89, respectively) were not significantly different (p = 0.13, Student´s t test = -1.61, df = 14), whereas the lowest and highest mean *He* (0.86 and 0.94) differed significantly (p = 0.0032, Student´s t test = -3.55, df = 14). Allelic richness (A_R_) and number of private alleles (A_P_) were lowest in the PS sample (10.33 and 1.4, respectively), while samples from SBC (13.23A_R_) and the NGC (4.23 A_P_) had significantly higher values (A_R_, p = 0.028, Student´s t test = -2.45, df = 14; A_P,_ p = 0.13 Student´s t test = -1.58, df = 14) (Table 1). LOSITAN detected five outlier loci in all comparisons among sample groups, but only two loci (MprB7 and MprA1-A11) were determined significant in comparisons between PS-PC and PS-NGC (Table F in S1 Table, supplementary material).

### Genetic differentiation

Power analyses indicated that the microsatellite data had could detect genetic differentiation as low as F_ST_ = 0.01 with 100% certainty, and with 93% and 95% certainty for F_ST_ = 0.001 using Chi-square and Fisher’s exact tests, respectively (Table G in S1 Table, supplementary material). Microsatellite and mDNA data were broadly concordant, revealing three or four genetic groups and consistently identifying barriers among them. Pairwise *ϕ*_*ST*_ values for mtDNA data were statistically significant among some sites for both SBC and NGC samples, but only the latter group was significantly differentiated from all other sites (Table 2). Pairwise sample F_*ST*_ comparisons for microsatellites indicated that costal hake stock extending from Washington State to Costa Rica (PC) was largely panmictic; the SBC sample showed some marginally significant differences from coastal samples, while those from PS and the NGC generally showed significant genetic differentiation from all other locations (Table 2).

**Table 2:** Pairwise F_*ST*_ values (below diagonal) and ϕ_*ST*_ (above diagonal) for *Merluccius productus*. Location abbreviations as in Table 1.

	PS	WS	OR	EU	SF	VIZ	SBC	NGC	CR
**PS**		0.142	0.028	-0.012	0.049	0.182*	0.147*	0.606*	0.492*
**WS**	0.035*		0.056	-0.106	0.027	0.002	0.180*	0.541*	0.393*
**OR**	-	-		-0.105	-0.014	0.090	0.126*	0.575*	0.401*
**EU**	0.028*	-0.003	-		-0.144	-0.063	0.039	0.488*	0.335
**SF**	0.041*	0.008	-	0.008		0.085	0.158	0.588*	0.451*
**VIZ**	0.035*	-0.001	-	-0.008	-0.001		0.103	0.450*	0.190
**SBC**	0.043*	0.012*	-	0.001	0.014*	0.002		0.512*	0.103
**NGC**	0.057*	0.029*	-	0.017*	0.031*	0.024*	0.032*		0.458*
**CR**	0.071*	0.017	-	0.009	0.014	0.012	0.015	0.019	

* significant after Bonferroni correction (*P* < 0.002).

The AMOVA analysis (Table 3) indicated that pooling locations geographically from PS to SBC, and assuming NGC and Costa Rica as different populations, produced the highest among-group variance (F_CT_ = 0.584) while minimizing variance within groups (F_SC_ = 0.602).

**Table 3:** Hierarchical analysis of molecular variance (AMOVA) of genetic variation of *Merluccius productus* for mitochondrial DNA (mtDNA) and microsatellite loci (M).

Structure	Source of variation	Percentage of variation		Fixation indices		*P* value	
		mtDNA	M	mtDNA	M	mtDNA	M
**A**. All localities	Among groups	41.56	2.75	0.416	0.027	<0.001	<0.001
	Within populations	58.44	97.25				
**B**. Three groups: Pacific coast–Puget Sound, northern Gulf of California, Costa Rica.	Among groups	58.45	2.1	0.584	0.021	0.028	0.108
	Among populations within groups	1.82	2.22	0.044	0.023	<0.001	<0.001
	Within populations	39.71	95.68	0.602	0.043	<0.001	<0.001
**C**. Three groups: Pacific coast, Puget Sound, northern Gulf of California.	Among groups	49.16	3.18	0.492	0.032	0.139	0.036
	Among populations within groups	5.96	0.55	0.117	0.006	<0.001	0.006
	Within populations	44.88	96.27	0.551	0.037	<0.001	<0.001
**D**. Four groups: Pacific coast, Puget Sound, northern Gulf of California, Costa Rica.	Among groups	50.25	3.4	0.502	0.034	0.059	0.017
	Among populations within groups	2.4	0.53	0.048	0.005	0.001	0.005
	Within populations	47.35	96.08	0.526	0.039	<0.001	<0.001

Pacific coast refers pooled locations from the Pacific Ocean (Washington, Oregon, Eureka, San Francisco, Vizcaino, Southern Baja California Peninsula).

**Fig 3:**
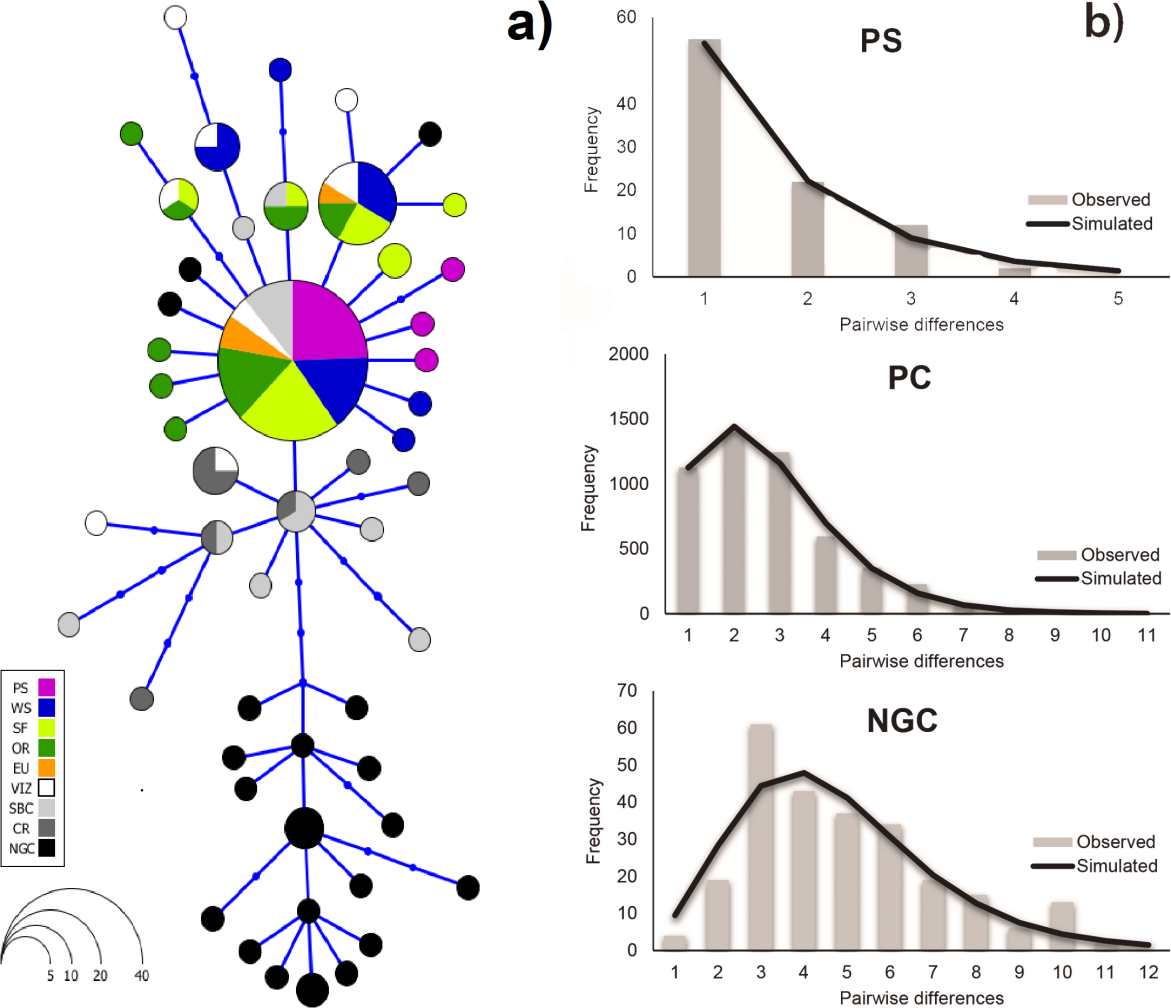
**Haplotype network (a) and nucleotide mismatch distribution (b) based on mtDNA data from *Merluccius productus*.** Abbreviations are as in Table 1.

A dominant haplotype (36% of total) was detected in all locations except the NGC and CR. The distinctiveness of hake from the NGC was strongly supported by a haplotype network with 17 private haplotypes separated by three mutational steps from all other locations (Fig 3A). Three haplotypes observed in CR were also present in SBC and VIZ but not elsewhere (Fig 3A).

**Fig 4:**
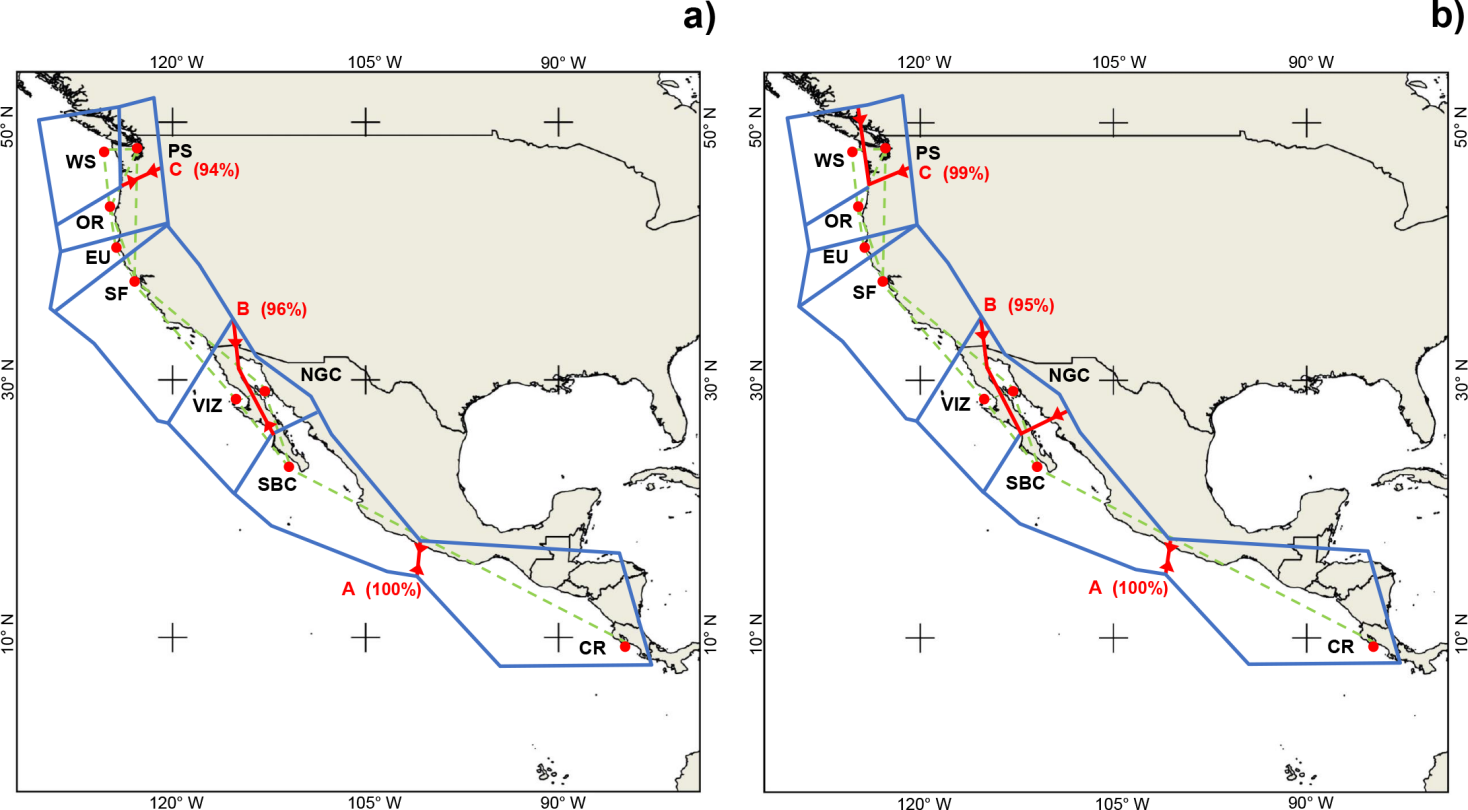
**Genetic barriers detected using pairwise population differentiation indices for *Merluccius productus*: a) mtDNA and b) microsatellite data.** Red lines indicate genetic barriers. Voronoï tessellation shown in blue and the corresponding Delaunay triangulation of samples in green. Numbers in parentheses indicate bootstrap percentages. Abbreviations are as in Table 1.

Barrier analysis of mtDNA data indicated three potential boundaries within the geographic distribution, all with high bootstrap support. The first was found between PS and the PC locations and the second between these coastal stations and the NGC. A third boundary separated CR from all other northern locations (Fig 4A).

AMOVA results for microsatellite data showed that genetic differentiation among groups was slightly higher when comparing four groups (D) PS, PC, NGC and CR (F_*CT*_ = 0.031; Table 3). Barrier analysis indicated three boundaries separating four groups (PS-PC, NGC-PC and CR-all other locations) with high bootstrap support for each (Fig 4B).

Over larger geographic scales, an isolation–by–distance analysis (IBD) pattern was evident in the microsatellite data (IBD test, *R*^*2*^ = 0.72, *P* = 0.008) (Fig A in S1 Fig, supplementary material). STRUCTURE assignment tests supported three population clusters (K = 3, ln P (D) = -16631.82, SD ± 5.9 and ΔK = 118.41): PS with 76% assignment, the NGC with 85% assignment and samples from the PC with 6% assignment (Fig 1). DAPC analysis produced three discriminant functions explaining 53.9% of the total variance, confirming the STRUCTURE results, but separated CR from the PC (Fig 1). Although estimated migration rates with microsatellites varied somewhat by runs (eleven runs), there was a pattern indicating higher rates between PC—PS than between PC—NGC (Fig B in S1 Fig, supplementary material). There was more migration from to NGC to PC than in the opposite direction and rates were roughly equal between PS—NGC.

### Historical demography

**Fig 5:**
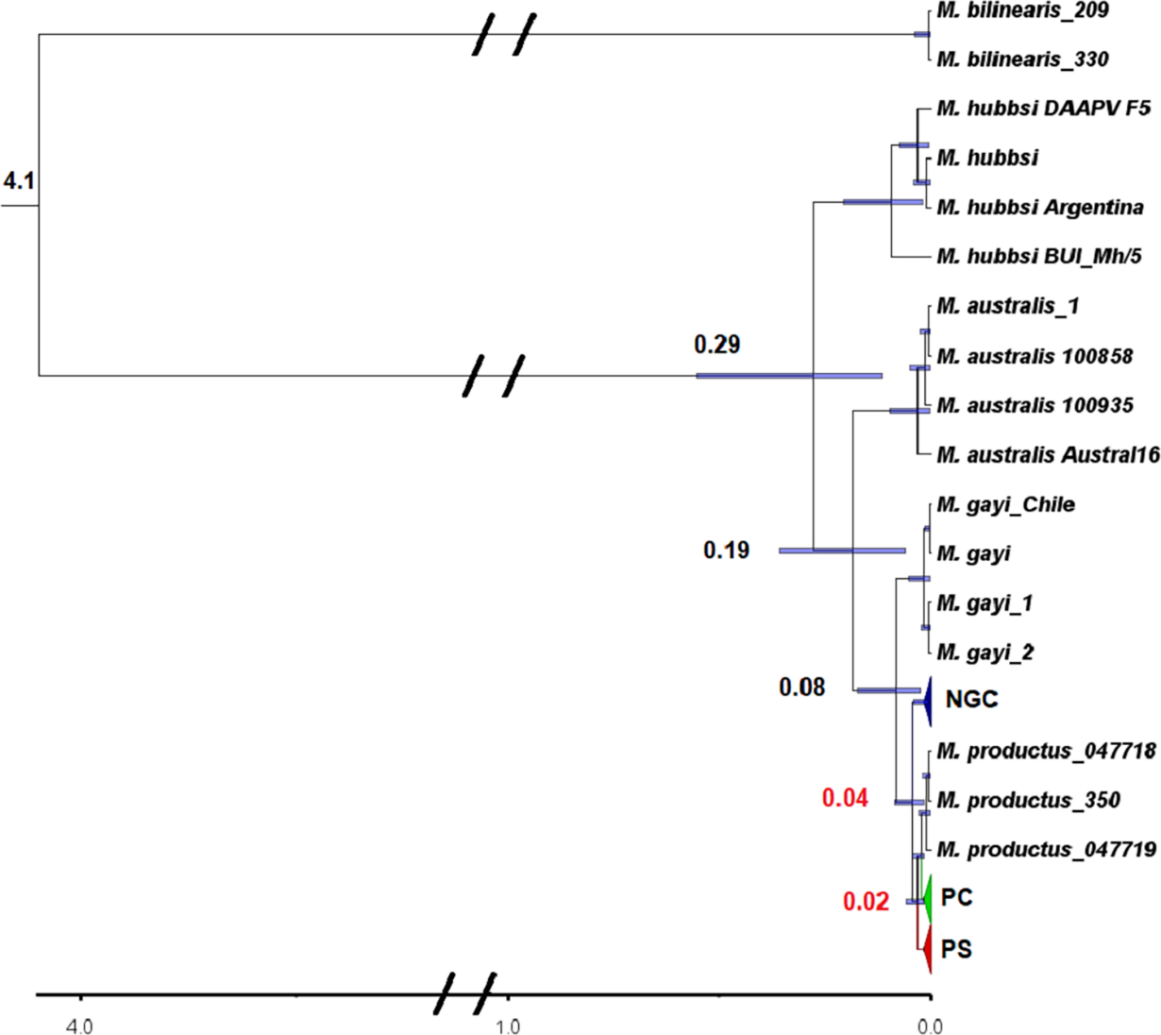
Relaxed Bayesian topology with the estimated divergence times based on CYTB sequences for populations of *Merluccius productus* obtained with BEAST [65]. Numbers at nodes indicate divergence time in millions of years. Blue bars correspond to the 95% highest posterior density (HPD) intervals. For clarity, samples from the NGC, PC and PS were collapsed into triangles.

Departures from neutrality were detected in both Tajima´s D and Fu´s Fs tests (Table 4). Values were significant and negative for all the three groups (PS, PC and NGC), suggesting possible historical population expansions, a demographic scenario further supported by unimodal mismatch distributions (Fig 3B). Low and non-significant raggedness index and SSD values (Table 4) showed a good fit between observed and expected distributions. The number of pairwise differences was higher in NGC, with peaks between 3–6 and a maximum of twelve mismatches. In the PC and PS samples, the greatest number of mismatches between the distributions were from one to three; however, the PC sample contained up to ten differences overall, while PS had only a maximum of five (Fig 3B). Divergence times estimated from BEAST were 18.5 kyr between PS and PC (95% highest posterior density interval 15.0–34.8 kyr) and 40 kyr between NGC and PC (95% highest posterior density interval 18.9–90.0 kyr) (Fig 5).

**Table 4:** Parameters of the mismatch distribution and neutrality test (Tajima’s *D*, Fu’s *Fs*) for populations of *Merluccius productus* based on mtDNA data.

Population / parameter	PS	PC	NGC
***t***	1.61	1.19	2.43
**SSD**	0.001	0.001	0.01
***P***_**SSD**_	0.81	0.84	0.31
**R**	0.16	0.02	0.04
***P***_**R**_	0.81	0.90	0.22
**Tajima's D**	-1.67	-2.34	-1.48
***P***_**D**_	0.03	0.00	0.05
**Fs**	-1.64	-27.60	-17.31
***P***_**Fs**_	0.02	0.00	0.00

Abbreviations are as in Table 1. Tau (*t*), sum of squared deviation (SSD), Probability (*P*), raggedness index (R), Tajima's value of selective neutrality (Tajima's D), Fu's value of selective neutrality (Fs).

### Frontal zones in the Gulf of California

#### Satellite data

February climatology data from the Aqua-MODIS satellite showed considerable differences in SSTd across the Gulf of California, ranging from ~ 25.5 ^o^C in the Gulf of California to ~17 ^o^C at the entrance of the Gulf (Fig 2). In particular, strong localized SST gradients were observed in the entrance of the Gulf, from ~ 26 ^o^C in the southern region to ~ 21 ^o^C in the north (Fig 2). The lowest SST was observed in the MAR (~ 17 ^o^C), showing frontal zones to the south and north.

#### Hydrographic data

**Fig 6:**
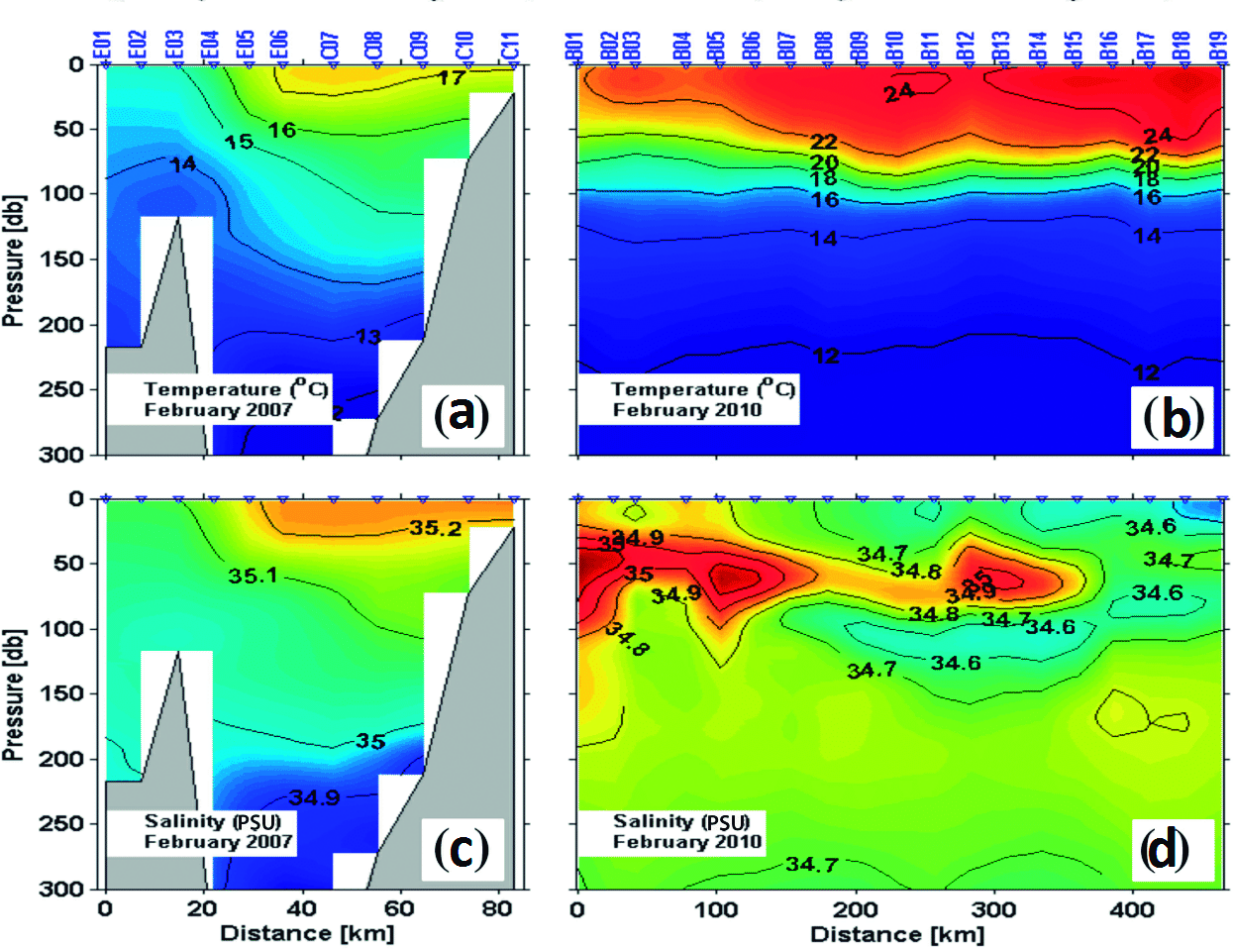
Temperature and salinity profiles. (a) and (c) depict temperature and salinity distributions across the front located south of the Midriff Archipelago Region (MAR). (b) and (d) depict temperature and salinity distributions across the entrance of the Gulf of California.

The transect crossing the frontal zone south of the MAR (Fig 6A and 6C) showed two distinct hydrographic domains: the northern part of the southern Gulf and the MAR. In the southern Gulf (stations E04-C11), thermal stratification was evident, ranging from 15°C at ~80 m to 17°C at the surface (Fig 6A). Salinity profiles were similar, increasing from ~35 PSU at 150 m to ~35.2 at the surface (Fig 6C). The water column was more homogeneous in the MAR domain (stations E01–E03), ranging from 13°C at ~ 200 m to 15°C at the surface (Fig 6A). A dome of 14 and 15°C isotherms defined the MAR frontal zone and the entrance to the Gulf as distinct hydrographic domains from the adjacent Pacific Ocean (Fig 6B and 6D). In the southern Gulf, thermal stratification was evident, ranging from 18°C at ~ 70 m depth to 22°C at the surface (Fig 6B, stations B01-B02). Maximum salinities in this area were from ~ 34.7 to 35.2 PSU (stations B01-B07) at 50–100 m depths, which extended into adjacent Pacific Ocean waters at thermocline depth and resulted in a strong subsuperficial salinity front (Fig 6B). The water column was more highly stratified in the Pacific Ocean stations B04-B19, ranging from 16°C at ~ 70 m depth to 22°C between 50–100 m. A minimum salinity (~ 34.6 PSU) was observed at stations B01-B14, extending from the thermocline to the surface (Fig 6D).

#### Seascape genetic analyses

**Fig 7:**
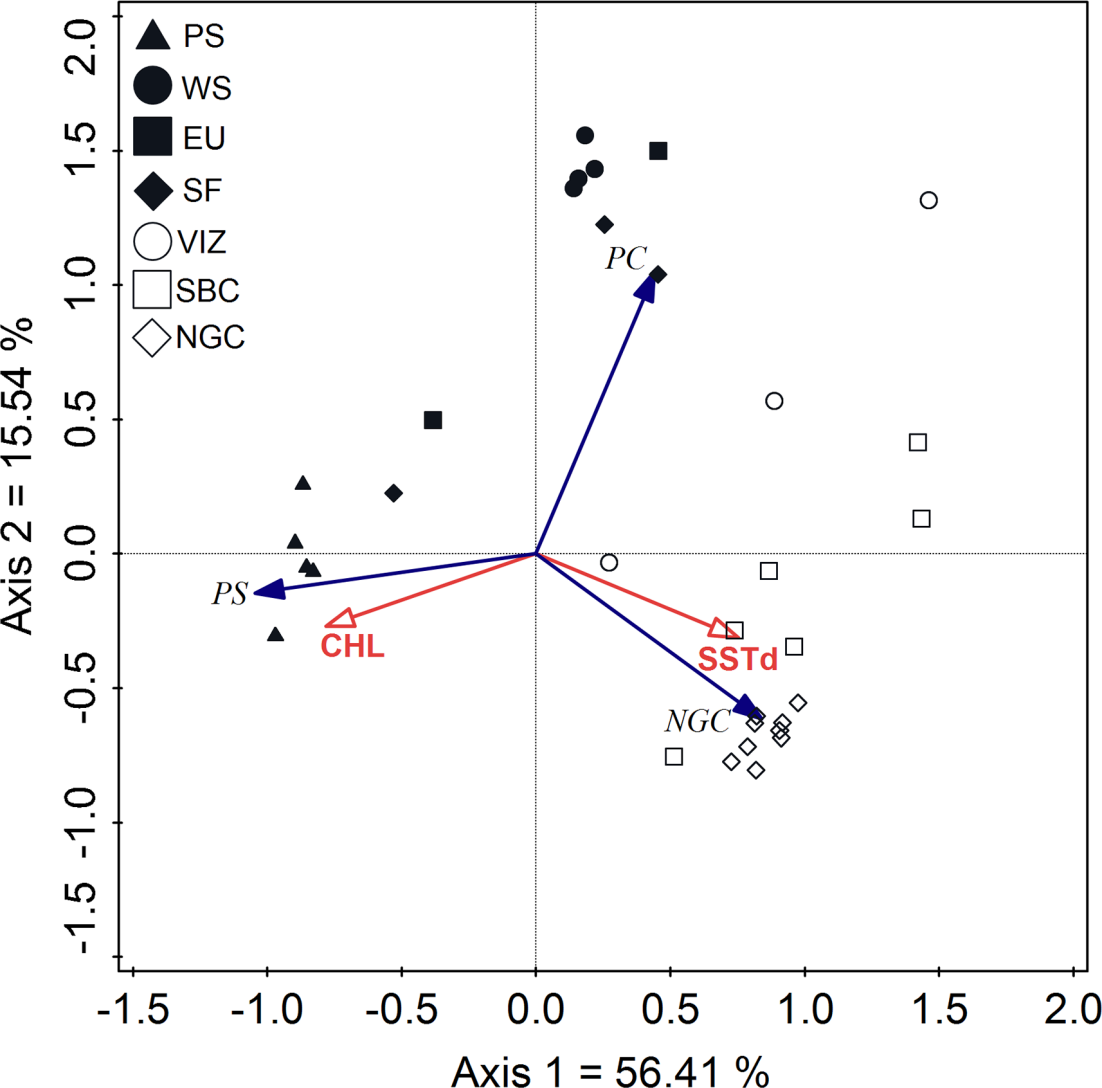
Redundancy analysis (RDA) for the association of oceanographic variables obtained from Ocean Color Web, and the ancestry coefficients of the three populations. Puget Sound (PS), Pacific Coast (PC) and Northern Gulf of California (NGC) generated in STRUCTURE. Chlorophyll *a* concentration (CHL) and sea day time surface temperature (SSTd).

Two oceanographic variables, SSTd and CHL, showed no significant autocorrelation (Table H in S1 Table, supplementary material) and were used in subsequent analyses. The first two axes of the RDA explained 71.95% of the total variance, suggesting that the three groups identified in genetic analyses (PS, NGC and PC) are separated due to opposing effects between SSTd and CHL (Fig 7). Both BIOENV and the DISTLM analyses confirmed those two variables as significant predictors, with SSTd having greater explanatory weight (BIOENV, SSTd, Rho = 0.98, P < 0.05, and CHL Rho = 0.98, P < 0.05, and both Rho = 1.0, P < 0.05; DISTLM, SSTd, 49.6%, and CHL 22.86%, and for both Rho = 0.725, P < 0.05).

## Discussion

### Genetic diversity and differentiation

The levels of genetic diversity observed with both molecular marker classes were comparable to those reported for other hake species. Mean haplotype diversity of *M*. *productus* in this study (0.86) exceeded those reported for *M*. *paradoxus* [11,79], *M*. *albidus* [28], *M*. *australis* [80], *M*. *bilinearis* [28] and *M*. *capensis* [11] and was equal to that reported for *M*. *gayi* [15]. Similarly, the eight microsatellites showed moderate to high levels of variation (*He* = 0.85 to 0.94), comparable to those reported for *M*. *merluccius* (0.70–0.96 [81–84]), *M*. *bilinearis* (0.88–0.91) and *M*. *hubbsi* (0.84 to 0.87) [85].

While it is unsurprising to find broad-scale panmixia or weak IBD structure in marine fishes with high dispersal capabilities, there is increasing genetic evidence that some gadoid species (cods and hakes) can colonize different ecosystems, allowing them to adapt and evolve independently [28,36]. In this study, we confirmed the presence of a widely panmictic stock along the eastern Pacific coast and two populations in partially isolated marginal seas documented in other studies [18–22] and found associations with oceanographic variables that may restrict gene flow among them. The coastal stock (PC) showed genetic homogeneity along a broad geographic range (approximately 3,250 km) in the Northeast Pacific Ocean. Our data revealed that the average ancestry coefficient was 0.62 in this group, indicating genetic admixture. The southern range of this population appears to share a larger ancestry component with the NGC stock, while the northern range appears to be admixed with PS (Fig 1). Our results also confirmed genetic differences over smaller geographical scales from the partially isolated waters of PS or NGC. This isolation is more evident in the NGC where there is an asymmetrical migration rate with the PC population (Fig B in S1 Fig, supplementary material). These results confirmed previous studies of *M*. *productus* and other hakes showing genetic structure indicating both migratory and resident population components [22,23,28,29]

However, there were some discrepancies displayed in the present study between the marker classes. The mtDNA analysis consistently revealed two genetic groups: one at NGC, which was differentiated from all other locations and the other encompassing all sites along the Northeast Pacific coast, including PS. The microsatellite data separated PS as a third distinct population. Estimated divergence times from CYTB sequences (Fig 5), suggest a more ancient separation (~ 40 kyr) of the NGC population from the coastal group (PC). Some observations support this hypothesis. First, the haplotype network showed 17 private haplotypes in the NGC separated by three mutational steps from the others and connected to those found only in the southern range (VIZ, SBC and CR). Secondly, mismatch analysis showed that NGC had a higher number of pairwise differences between observed and predicted distributions compared to samples from the PC and PS. Finally, the estimated expansion date of this population was earlier than for the divergence between PC and PS (Table 4). Together these results suggest that NGC hake is a relict population.

Pacific hake originated from an ancestor similar to *M*. *bilinearis* (Atlantic hake) or another apparently extinct species that entered the Pacific Ocean at the closing of the isthmus of Panama, subsequently speciating into the northern (*M*. *productus*) and southern hake (*M*. *gayi*) about 1 myr ago in the Mid-Pleistocene [67,86]. However, these estimates were derived from allozyme polymorphisms or CYTB sequences assuming a constant rate of molecular evolution. Our analysis indicates heterogeneity of nucleotide substitution rates of CYTB among some species (Table I in S1 Table, supplementary material). In addition, our estimates of divergence times were made with contemporary calibrations of known events, hence some estimates reported in the literature may be overestimated. Our results indicate that divergence of *M*. *asutralis* and the clade of Pacific hake ancestral to *M*. *gayi* and *M*. *productus* was recent (290 kyr) and that of *M*. *gayi* and *M*. *productus* exceptionally so (80 kyr: Fig 5 and Table J in S1 Table, supplementary material). If our estimates are correct, contemporary *M*. *merluccius* populations have diverged since the late Pleistocene or Holocene and NGC hake is a relict population barely younger than its southern Pacific relative, *M*. *gayi*.

The Gulf of California is a semi-enclosed marginal sea that has been extensively studied to assess the degree of isolation in other marine species inside the Gulf relative to the Northeast Pacific Ocean [12,87–90]. Two widely debated hypotheses have been proposed to explain genetic isolation in marine fauna [91,92], one contingent upon the highly dispersive capacities of temperate species during Pleistocene glaciation [92] and another that invokes vicariant events, such as the sea ways along of Peninsula of California [12,87,93]. Our divergence time estimates clearly indicate that the genetic structure of North Pacific hake results from more recent temporal events, such as post-Pleistocene glaciation period (Fig 5). The estimate for the separation of PS and PC populations, approximately 15.5 kyr, are concordant with those estimated for coastal versus Salish Sea (including Puget Sound) Pacific cod, *Gadus macrcephalus*, indicating post-glacial expansion into the region [36].

Microsatellite results indicate at least three genetically discrete populations exist: two resident stocks (NGC and PS) and the contiguous Pacific coast from Washington State to Costa Rica (Fig 1). Results for the CR sample are arguably inconclusive, although the power analyses indicated sufficient power to detect genetic significant differentiation at very low F_ST_ values (0.001). The small sample size (8 individuals) from CR produced estimates greater than that but without statistical support. The SBC population (dwarf stock) was considered as an independent stock [94] but our analyses derived from neutral markers failed to clearly identify that and these hake showed only marginal statistically significant values of genetic differentiation (Table 2). Further research is required to confirm the genetic distinctiveness of CR and dwarf stock (SBC) populations.

### Role of oceanography

Studies reporting mesoscale oceanographic features as causal agents for population isolation in the Gulf of California rarely provide concurrent oceanographic data to explain observed genetic structure in different marine species [31,33,34,95]. Others have used simulations of particles as a proxy for the effects of circulation on creating these patterns [95,96]. Here, we contribute data to support the hypotheses that oceanographic barriers contribute to isolating resident populations of North Pacific hake.

The efficacy of barriers to gene flow between species with different life history strategies (dispersal ability, reproductive strategy, habitat specialization, etc.) has been extensively studied inside Gulf of California. Multiple patterns of genetic structure have been reported, including a) panmixia [32], b) IBD along the Gulf of California [13], c) genetic differentiation between north and south [95], d) between east and west [13] and e) between the Gulf of California and the Pacific Ocean [12,31,33,34,97]. These studies suggest that putative barriers to gene flow do not function in the same way across the diverse life histories exhibited by marine organisms.

Previous oceanographic surveys [98,99], studies of larval fish ecology [100] and results from this study indicate two mechanisms that could promote isolation in the Gulf of California region: permanent temperature and salinity frontal zones in the MAR and the strong salinity and temperature gradients in the entrance of the Gulf of California [98,99, 100]. One of the most persistent oceanographic features is observed around the MAR (Fig 2), where an area of minimum sea surface temperature (SST) occurs due to intense tidal-mixing and convergence-induced upwelling in the Ballenas Channel [101,102]. This minimum SST area is delimited to the south and, with lower intensity, to the north, by temperature fronts that frequently show eddies and filaments surrounding the MAR [103]. Danell-Jiménez et al. [100] and Inda-Diaz et al. [102] examined vertical distributions of fish larvae across the fronts during summer and winter and reported that abundance for most species decreased considerably across the front and, for some other species, the fronts constitute a barrier for larval fish dispersal northward. The circulation in the NGC, which reverses seasonally (anticyclonic in winter and cyclonic in summer [98,99]), could be another physical barrier. Sánchez-Velasco et al. [104] reported that NGC hake larvae are concentrated in the winter anticyclonic eddy in the northern Gulf. The absence of hake larvae between the southern edge of the MAR and the entrance to the Gulf of California (LSV, personal communication) infers a high potential for isolation of this resident population at this life history stage. Other processes potentially influencing the isolation of NGC hake are hydrographic gradients resulting from the confluence of surface water masses in the entrance of the Gulf of California (Fig 6). In this region, a complex thermohaline structure is driven by the confluence of Tropical Surface Water, subsurface California Current Water and Gulf of California Water [98,105]. The coincidence of these oceanographic features with the geographic barrier detected in our analysis (Fig 4), strongly suggests their effects as drivers of genetic isolation in Pacific hake.

### Seascape genetics

Multiple studies have reported significant genetic–environmental association in marine fish [4,11,106]. The seascape genetics approach taken here assessed the relationships for SST and chlorophyll in populations of hake from PS and NGC. There was a clear inverse relationship between SST and CHL concentration, with more northern sites (PS, WS, EU and SF) being colder and having higher CHL concentrations than southern ones (VIZ, SBC and NGC). PS and NGC both have higher values of CHL than the coastal zone of the Pacific (Fig 7). The PS environment is colder with higher concentrations of CHL, not uncommon in fjords with cooler freshwater inputs from adjacent basins (S1 Text, supplementary material).

Although we found a significant genetic–environment association between PS and NGC hake, the explanation of how these variables keep resident stocks isolated is not simple. We interpreted results from two marker classes (mitochondrial and nuclear) in a neutral population genetics framework. The mitogenome is generally assumed to evolve under neutral or nearly neutral selection [107]. Recent evidence, however, indicates that the assumption of neutrality is frequently violated [108]. In a study with locally adapted Atlantic salmon populations distributed along a latitudinal cline in northern Atlantic Ocean, Consuegra et al. [109] found evidence of positive selection in the Artic populations, suggesting adaptation to low temperatures. In our study, using a concatenated fragment of CYTB and COI and 16S sequences, we found evidence of deviations from neutral expectation in hake from NGS and PC (Table 4). Seventeen private haplotypes (Fig 3) and inferred insolation since at least the late Pleistocene support this premise (Fig 5). Nonetheless, more detailed studies, similar to those performed by Consuegra et al. [109] and Blier et al. [110], are necessary to confirm a hypothesis of localized adaptation in *M*. *productus*. An alternative explanation is that physical barriers that impede genetic flow, such as gradients and oceanographic fronts, are the sufficient to create the observed genetic differentiation of populations living in local heterogeneous environments.

Two microsatellite loci, MprB7 and MpA1-11, were detected as F_ST_ outliers in some comparisons among the genetic groups (Table F in S1 Table, supplementary material). Outlier loci are common in both coding and non-coding regions of the genome, often representing a substantial fraction of the loci (2–10%, [111]). Thus, the occurrence of outlier loci in this study is not convincing evidence for the effects of selection. Alternative explanations have been proposed [112]; one is the coupling of endogenous and exogenous barriers, in spatially subdivided populations. Endogenous barriers may form, due to incompatibilities between groups of alleles through underdominance, epistasis or pre-zygotic isolation, and exogenous barriers may result from groups of alleles adapted to different environments. In our study, outlier loci likely originated from genetic substructure rather than from selection.

A possible ad hoc explanation for the findings of significant genetic-environmental association could be related with the habitat and feeding of larvae and juveniles of the hake and the upwelling phenomenon. Oceanographic data, averaging four years (2006–2009) and four months (March, April, May and June), were selected for coincidence of hake on the spawning grounds and duration of the planktonic larval stage. Hake are dependent upon upwelling ecosystems (S1 Text, supplementary material), a common feature in Eastern Boundary Current Systems in both the northern and southern Atlantic and Pacific oceans. Nearshore upwelling zones are among the most productive fishing areas in the world [113] and support large biomasses of small planktivorous pelagic fish (small pelagics) through high levels of primary and secondary productivity in these regions [114]. Sea surface temperatures and CHL concentrations are related to changes in spawning areas [115]. Since zooplankton biomass is higher in colder waters compared to warmer ones, a match between spawning timing and subsequent larval transport to areas of high food concentration may be crucial for successful recruitment [116]. In addition, euphausiids are a primary item in hake diets and their abundance is closely linked to upwelling and high primary production [108,117,118]. Natal or spawning site fidelity in hake could also reinforce genetic isolation. This reproductive behavior has been reported in related gadoid species that show comparable genetic structure to Pacific hake [15,28,84,119–122], but further research is needed to resolve factors contributing to genetic isolation of Pacific hake.

### General considerations

Pacific hake is considered a potential fishery resource in Mexican water [123]. Results presented here are potentially useful for identifying conservation units, determining levels of population connectivity and developing harvest and conservation management strategies [124–126]. Since 2012, Mexico started fishing for hake from the Gulf of California conducted largely during the spawning period (http://cobi.org.mx/wp-content/uploads/2013/10/Reporte-Merluza-lowres-200913.pdf). North Pacific hake from the NGC should be considered a distinct population for assessment and management purposes, since oceanographic barriers in this region likely create some degree of isolation, as indicated for PS hake in the Salish Sea ecosystem [22,23]. The geographic areas that the NGC and PS populations occupy may thus represent important conservation units, serving as potential refuges for global genetic diversity of hake and other marine species [12,88,89]. In addition, the diversity of genetically heterogeneous populations (portfolio effect) contributes to long-term sustainability, resilience and productivity of species within these ecosystems [127]. The current controversy over whether to recognize the NGC hake population as a distinct species, *M*. *hernandezi* [24–26] suggests species–level divergence in this population. In this sense, North Pacific hake may also represent a good model for understanding the early stages of allopatric speciation [12], since at least two isolations (PS and NGC) have arisen independently at different times in the Northeast Pacific Ocean.

## Supporting information

S1 Table Table A. Collection dates and geographical coordinates of sampling sites. Table B. Genbank accession numbers for CYTB sequences of *Merluccius* spp. used to estimate divergence times. Table C. Estimates of null allele frequencies using the FreeNA program (Chapuis & Estoup 2007). Table D. Test of Hardy-Weinberg disequilibrium by locus. A. Probability test. B test when H1 = Heterozygote deficit. Significant values (in red) after Bonferroni correction. Table E. Linkage disequilibrium test. P-value for A pairwise comparison of locus by location and B locus pairs across all locations. Table F. Loci under selection estimated by the random value method F_ST_. Table G. Statistical power analysis for microsatellite data to detect genetic differentiation by using POWSIM v 4.0 (Ryman & Palm 2006) [51]. Proportion of significances (P<0.05) for summed /combined test statistics. Fisher’s exact test and traditional Chi-square. Table H. Spearman rank correlation coefficients for oceanographic variables. SSTd (day surface temperature), SSTn (night surface temperature), PAR (Photosynthetically Available Radiation), POC (Particulate Organic Carbon), water turbidity (measured as a diffusion attenuation coefficient at 490 nm—KD490, nm), and CHL (Chlorophyll a concentration, mg/m3). Diagonal lower Rho values, diagonal upper p values. Pairwise values of Rho considered strongly auto-correlated are highlighted in yellow. Table I. Test of the homogeneity of substitution patterns between sequences of CYTB. The probability of rejecting the null hypothesis that sequences have evolved with the same pattern of substitution, as judged from the extent of differences in base composition biases between sequences (Disparity Index test, [1]). A Monte Carlo test (10000 replicates) was used to estimate the P-values [1], which are shown above the diagonal. P-values smaller than 0.05 are considered significant (marked with yellow highlights). The estimates of the disparity index per site are shown for each sequence pair below the diagonal. The analysis involved 41 nucleotide sequences. All positions containing gaps and missing data were eliminated. There were a total of 262 positions in the final dataset. Disparity index per site are shown for each sequence pair below the diagonal. The analysis involved 41 nucleotide sequences. All positions containing gaps and missing data were eliminated. There were a total of 262 positions in the final dataset. Evolutionary analyses were conducted in MEGA6 [2]. 1. Kumar S. and Gadagkar S.R. (2001). Disparity Index: A simple statistic to measure and test the homogeneity of substitution patterns between molecular sequences. Genetics 158:1321–1327. 2. Tamura K., Stecher G., Peterson D., Filipski A., and Kumar S. (2013). MEGA6: Molecular Evolutionary Genetics Analysis version 6.0. Molecular Biology and Evolution30: 2725–2729. Table J. Estimated divergence times based on CYTB sequences for populations of *Merluccius productus* obtained with BEAST [65]. Numbers in bold indicate divergence times and numbers in parentheses correspond to the 95% highest posterior density (HPD) intervals.
(XLSX)

S2 Table Microsatellite database. (XLSX)

S1 Text Description of the ecosystems inhabiting the hake in the Northeast Pacific. (DOCX)

S1 Fig **Fig A.** Isolation by distance relationship. Scatter plot of pairwise genetic distance (linearized F_ST_) *vs* geographic distances of eight microsatellite loci for *Merluccius productus* showing significant correlation between geographic and genetic distance. **Fig B.** General patterns of estimates of migration rates between *Merluccius productus* populations. A. Average estimates of migrants per generation with range in parentheses. Arrows indicates direction of gene flow. B. Depiction of migration rates, with line thickness proportional to migration rate. PC Pacific coast, PS Puget Sound and NGC northern Gulf of California.
(DOCX)
