# Supplementary material for: Role of oceanography in shaping the genetic structure in the North Pacific hake Merluccius productus
Source: PLoS One. 2018 Mar 26;13(3):e0194646. doi: 10.1371/journal.pone.0194646 (PMC5868808; doi:10.1371/journal.pone.0194646)
Supplement: S1 Text — (DOCX) [file pone.0194646.s003.docx]

**S1. Text**

**Description of the ecosystems inhabiting the hake in the Northeast Pacific**

**California Current Ecosystem (CCE)**

For a more complete description of the system, see Benson (2002) and King et al. (2011). Here we summarize aspects of the ecosystem where the coastal stock of Pacific hake, *Merluccius productus*, occurs. The CCE is an upwelling system that extends from Baja California to the northern tip of Vancouver Island. There are three principal large –scale oceanic circulation features the dominant year-round equatorward California Current (CC), the poleward California Undercurrent (CU) and the seasonal poleward Davidson Current (DC) along the continental slope. The CC is a slow, shallow subarctic flow characterized by low salinity and temperature and nutrient-rich water from the North Pacific. The CU flows northward along the continental slope carrying warm, high-salinity, low oxygen water from Baja California to at least Vancouver Island. In winter, a surface poleward flow (DC), originates close to Pt. Conception (~35°N) and travels northward past Vancouver Island. In the northern and coastal regions, upwelling during spring and summer contributes to additional cooling of surface (and possibly deep) waters due to the influence of subarctic water. The southern and offshore regions, dominated by subtropical water, show little annual variability of the thermocline, with reduced stratification in summer and autumn in the mixed seasonal layer.

The variability of the current California ecosystem is influenced by phenomena that occur over different temporal scales (seasonal, interannual and decadal), and with the El Niño Southern Oscillation, which, together with atmospheric pressure systems, affect sea surface height, temperature, intensity of upwelling, and the presence of eddies, jets, etc. Some authors have shown that eddies are true oasis for higher trophic marine life (Godø et al. 2012).

Seasonal variation in primary production in the CCE can be divided into three periods based on prevailing wind patterns: Northwesterly winds dominate the upwelling season (February–September), allowing nutrient-rich water to ascend and support increased phytoplankton production inshore. The short oceanic season (September–October) is characterized by decreased wind, effectively stopping upwelling. During this period, primary production rates decrease. Southerly winds dominate in winter (November–February) creating t downwelling, where oceanic water flows into the coastal region following a poleward path along the continental shelf. Chlorophyll varies with latitude, being approximately twice as high in the northern CCE as in southern areas (Ware and Thomson 2005).

Migratory Pacific hake coastal stock

Pacific hake (migratory stock) is the most abundant commercial fish off the west coast of North America; for this reason, the biology and life history of this stock have been studied in detail, see Benson (2002) for review. This population migrates annually between spawning grounds from central California to the Baja California Peninsula in winter to feeding grounds from central Oregon to northern Vancouver Island in summer (Saunders and McFarlane 1997). Although the spawning ground in CCE are not well defined because spawning schools have not been observed (Bailey et al. 1982), ninety-five percent of spawning takes place between mid-December and early March (Smith 1995). Larvae occur in two areas: one of high abundance along the coast from San Francisco southward to north of Baja California and another one of lesser abundance in the southern part of Baja California (Moser et al. 1993). The latter is as attributed to the Pacific hake dwarf stock (Funes-Rodriguez et al. 2009) which, according to Balart (2005), spawn occur between March and May.

Anomalous climatic or oceanographic conditions have profound effects on the recruitment of populations during the multiple life-history stages of Pacific hake (Hollowed 1992; Phillips et al. 2007). For example, high temperatures, reduced southerly flow of the CC, weak upwelling and poleward currents could transport eggs and larvae away from favorable nursery areas (Bailey 1981; Funes-Rodríguez et al. 2009) or displace spawners from normal spawning areas in the CCE (Agostini et al. 2007). The success of hake recruitment in the CCE depends on the transport of larvae and the abundance of prey. Cold years with intense upwelling results in reduced growth rates, low prey abundance and high predation mortality (Bailey and Francis 1985; Hollowed and Bailey 1989). During warmer years (e.g. El Niño), the intensity of upwelling is reduced, which may favor larval survival (Smith et al. 2001). Eddies (both offshore and nearshore), jets and meanders in the CCE are also areas of highest larval survival, possibly due to increased availability of prey and larval retention in favorable habitats (King et al. 2011). Thus, circulation features of upwelling regions seem to be consistently related to the recruitment of hake throughout the northeastern Pacific. The diet of Pacific hake is strongly dependent on upwelling systems, since juveniles prefer large copepods and euphasiids and the adults are active predators preying on small pelagics that inhabit upwelling zones (Pitcher and Alheit 1995). For these reasons and, like other hake species, it is considered to have life-history characteristics specialized for upwelling zone hábitats (Benson 2002).

**Puget Sound ecosystem**

The Puget Sound is part of the Salish Sea ecosystem, which extends inland along the US-Canada border. This fjord ecosystem is influenced by fresh water inputs from rivers in surrounding watersheds (Quinn 2010). Nevertheless, the topography and bathymetry of the ecosystem was greatly transformed during the Wisconsin Glacial Episode, which included three major continental glaciations beginning about 70,000 years ago and separated by relatively warm interglacial periods (Quinn 2010). Puget Sound was largely ice-free by about 15 kya (Menounos et al. 2008). During these repeated glaciations, ice-free zones could serve as glacial refuges that provided sources of colonizers during interglacial periods (McPhail and Lindsey 1986) and the fragmentation of habitats promoted genetic divergence in isolation (Waples et al. 2008). For these reasons, it has been hypothesized that Puget Sound hake was isolated from the large coastal stock just after the last glacial retreat~ 15 kya (Iwamoto et al. 2004; King et al. 2012; Chittaro et al. 2013).

Tidal currents dominate water circulation in Puget Sound. The deep, denser coastal waters enter Puget Sound from the Juan de Fuca Canyon through Admiralty Inlet and lower salinity outﬂowing surface water from rivers ﬂushes Puget Sound. The outflow rate of Puget Sound is dictated in part by the relative magnitudes of these two processes. Net transport of nutrients from Juan de Fuca Canyon generates high productivity inside the Puget Sound (Martin and MacCready 2011; Matthew and MacCready 2014). A phytoplankton bloom results, usually beginning around April or May and ending in the summer (McCready & Banas 2016). Winter et al. (1975) state that the high productivity typical of Puget Sound is due to strong, persistent upwelling of nutrients and algal cells from depth.

Resident Pacific hake in Puget Sound

In Puget Sound, the resident population of hake has declined dramatically in the past three decades, leading to a closure of the fishery in 1990 (Gustafson et al. 2000) and a designation by NOAA Fisheries as a Species of Concern in 1999. These hake are classified as part of the Georgia Basin Distinct Population Segment (DPS) (Iwamoto et al. 2015), which is considered discrete from the highly-migratory coastal stock. It is also evident that the preferred habitat of this resident stock is in areas of upwelling and eddies. The decline of this trophic level component has important implications for the functioning of the Puget Sound ecosystem.

Seasonal levels of maximum primary productivity in Puget Sound match partially with the spawning period of hake from February through April in Port Susan (Pedersen 1985) and Dabob Bay (Bailey and Yen 1983) in Puget Sound. The Puget Sound hake are related to the upwelling and eddies that occur within the estuarine ecosystem, where they seek the availability of food and survival of their larvae as has been described in the migratory stick in the CCE (King et al. 2011).

**Northern Gulf of California**

The Gulf of California is the only semi-enclosed sea in the Eastern Pacific (Brusca et al. 2017), it is characterized by seasonality in the near-surface thermohaline properties and water-column structure but also in circulation patterns: cyclonic in summer and anticyclonic in winter (see review by Lavín and Marinone 2003). Salinities in the Gulf have always been higher than in the adjacent Pacific Ocean at the same latitude. It is one of the most productive oceanic ecosystems (Álvarez-Borrego 2010).

Permanent oceanographic features occur in the mouth of the Gulf of California, where differentiated CCE and the Tropical-Subtropical water masses converge (Portela et al. 2016). The temperature and salinity differences between these water masses create strong hydrographic gradients and permanent fronts, which tend to develop mesoscale structures such as eddies, jets, and meanders (Lavín et al. 2004). These features may limit entry into the Gulf of California for some adult temperate fish species or limit dispersal of their eggs and larvae (Davies et al. 2015). Inside the Gulf of California, a distinct area of low sea surface temperature resulting from intense tidal-mixing and convergence-induced upwelling in the Ballenas Channel permanently surrounds the Midriff archipelago region (MAR) (Inda-Diaz et al. 2010; López et al. 2014). This frontal area frequently has eddies and filaments (Navarro-Olache et al. 2004) that, although poorly documented, potentially restrict the dispersal of fish species. Connectivity studies in this region report that reversible eddies concentrate planktonic organisms in the northern region (NGC), where larvae of *M. productus* have been associated with the anticyclonic eddies produced in winter (Sánchez-Velasco et al. 2009; Santiago-García et al. 2014). These hydrographic gradients and fronts appear to function as natural barriers to genet flow, causing genetic isolation of stocks of fishes such as Pacific hake.

NGC stock

In general, very little is known about the biology of NGC hake. The seasonal occurrence of larvae in only the northern region, suggests that local retention may be high due to winter anticyclonic eddies (Sánchez-Velasco et al. 2009; Santiago-García et al. 2014). In addition, larvae have not been observed in the central and southern part of the Gulf of California, suggesting that spawning occurs only in the NGC. Spawning is estimated to occur from November to April, with maximum intensity in January and February (Sánchez-Velasco et al. 2009). This population has been previously shown to be genetically different from the coastal stock found in the Pacific Ocean (Iwamoto et al. 2015).

References

Agostini VN, Hendrix AN, Hollowed AB, Wilson CD, Pierce SD and Francis RC. 2007. Climate–ocean variability and Pacific hake: A geostatistical modeling approach. Journal of Marine Systems. 71(3–4). 237-248.

Álvarez-Borrego S. 2010. Physical, chemical, and biological oceanography of the Gulf of California. In: Brusca GC (ed.), The Gulf of California: Biodiversity and Conservation. University of Arizona Press, Tucson, pp. 24–48.

Bailey KM. 1981. Larval transport and recruitment of Pacific hake *Merluccius productus*. Marine Ecology Progress Series 6, 1–9.

Bailey KM, Francis RC and Stevens PR. 1982. The life history and fishery of Pacific whiting, *Merluccius productus*. Calif Coop Ocean Fish Invest Rep 23:81–98

Bailey KM and Francis RC. 1985. Recruitment of Pacific whiting, *Merluccius productus*, and the ocean environment. Marine Fisheries Review, 47: 8–14.

Bailey, K. M., and Yen, J. 1983. Predation by a carnivorous marine copepod, Euchaeta elongate Esterly, on eggs and larvae of the Pacific hake, *Merluccius productus*. Journal of Plankton Research, 5: 71–82.

Balart-P EF. 2005. Biología y ecología de la merluza de Bajacaliforniana, *Merluccius angustimanus* Garman, 1899, en la costa occidental de Baja california Sur, México. PhD Thesis, Universidad Autónoma de Nuevo León, México. 172 p.

Benson AJ. 2002. Oceanographic influence on Pacific hake (*Merluccius productus*) distribution and biology. Master of Science thesis. University of British Columbia. 134p.

Brusca RC, Álvarez-Borrego S, Hastings PA, Findley LT. 2017. Colorado River flow and biological productivity in the Northern Gulf of California, Mexico. Earth-Science Reviews 164 (2017) 1–30.

Chittaro PM, Zabel, RW Palsson W and Grandin C. 2013. Population interconnectivity and implications for recovery of a species of concern, the Pacific hake of Georgia Basin. Mar Biol (2013) 160:1157–1170.

Davies SM, Sánchez-Velasco L, Beier E, Godínez VM, Barton ED, Tamayo A. Three-dimensional distribution of larval fish habitats in the shallow oxygen minimum zone in the eastern tropical pacific ocean off Mexico. Deep Sea Res Part I Oceanogr Res Pap. 2015;101:118–29.

Funes-Rodríguez R, Elorduy-Garay JF, Hinojosa-Medina A and Villafranco Z. 2009. Interannual distribution of Pacific hake *Merluccius productus* larvae in the southern part of the California Current. Journal of Fish Biology (2009) 75, 630–646.

Godø OR, Samuelsen A, Macaulay GJ, Patel R, Hjøllo SS, Horne J, Kaartvedt S, Johannessen JA. 2012. Mesoscale eddies are oases for higher trophic marine life. Plos One. 7 (1). e30161

Gustafson RG, Lenarz WH, McCain BB, Schmitt CC, Grant WS Builder TL, Methot RD. 2000. Status review of Pacific Hake, Pacific Cod, and Walleye Pollock from Puget Sound, Washington. U.S. Dept. Commer. NOAA Tech. Memo. NMFS-NWFSC- 44, 275 p

Hollowed AB and Bailey KM 1989. New perspectives on the relationship between recruitment of Pacific hake and the ocean environment. In Effects of Ocean Variability on Recruitment and an Evaluation of Parameters Used in Stock Assessment Models, pp. 207–220. Ed. by Beamish RJ and G A McFarlane. Canadian Special Publication of Fisheries and Aquatic Sciences, 108.

Hollowed AB. 1992. Spatial and temporal distributions of Pacific hake, *Merluccius productus,* larvae and estimates of survival during early life stages. California Cooperative Oceanic Fisheries Investigations Report 33, 100–123.

Inda-Diaz EA, Sanchez-Velasco L, Lavín MF. Three-dimensional distribution of small pelagic fish larvae (*Sardinops sagax* and *Engraulis mordax*) in a tidal-mixing front and surrounding waters (Gulf of California). J Plankton Res. 2010;32(9):1241–54.

Iwamoto E, Ford MJ, Gustafson RG. Genetic Population Structure of Pacific Hake, *Merluccius productus*, in the Pacific Northwest. Environ Biol Fishes. 2004;69(1–4):187–99.

Iwamoto EM, Elz AE, García-De León FJ, Silva-Segundo CA, Ford MJ, Palsson WA, et al. Microsatellite DNA analysis of Pacific hake *Merluccius productus* population structure in the Salish Sea. ICES J Mar Sci. 2015; 72(9), 2720-2731.

King JR, Agostini VN, Harvey CJ, McFarlane GA, Foreman MGG, Overland JE, Lorenzo ED, Bond NA, and Aydin KY. 2011. Climate forcing and the California Current ecosystem. ICES Journal of Marine Science (2011), 68(6), 1199–1216.

King JR, MacFarlane GA, Jones SRM, Gilmore SR and Abbott CL. 2012. Stock delineation of migratory and resident Pacific hake in Canadian waters. Fisheries Research 114: 19-30.

Lavín MF and Marinone SG. 2003. An overview of the physical oceanography of the Gulf of California. In: Velasco O, Sheimbaum J, Ochoa JJ (Eds.), Nonlinear processes in geophysical fluid dynamics. Kluwer Academia Publishers, Dordrecht, The Netherlands, pp. 173–204.

Lavín MF, Castro R, Beier E, Godínez VM, Amador A, Guest P. SST, thermohaline structure, and circulation in the southern Gulf of California in June 2004 during the North American Monsoon Experiment. J Geophys Res. 2009;114(C2):1978–2012.

López M, Candela J, Argote ML. Why does the Ballenas Channel have the coldest SST in the Gulf of California? Geophys Res Lett. 2006; 33(11):L11603.

Matthew HA and MacCready P. 2014. Flow admixing in Juan de Fuca Canyon, Washington. Geophys. Res. Lett., 41, 1608–1615

MacCready P and Banas N. 2016. Linking Puget Sound primary production to stratification and atmospheric drivers on seasonal to inter-decadal scales. SSMSP Technical Report (http://marinesurvivalproject.com/wp-content/uploads/MacCready-Banas-2016-Tech-Rept.pdf).

Martin WD and MacCready O. 2011. Influence of large-scale tidal asymmetry on subtidal dynamics in the western strait of Juan de Fuca, J. Geophys. Res., 116, C02009

McPhail JD and Lindsey CC. 1986. Zoogeography of the freshwater fishes of Cascadia (the Columbia system and rivers north to the Stikine). In Hocutt CH, and Wiley EO, eds. The Zoogeography of North American Freshwater Fishes, pp. 615–637. John Wiley and Sons, New York, USA.

Menounos B, Osborn G, Clague JJ, Luckman BH. 2008. Latest Pleistocene and Holocene glacier fluctuations in western Canada. Quaternary Science Reviews, 28, 2049–2074.

Moser HG, Charter RL, Smith PE, Ambrose DA, Charter SR, Meyer CA, Sandknop EM and Watson W. 1993. Distributional atlas of fish larvae and eggs in the California Current Region: taxa with 1000 or more total larvae, 1951 through 1984. California Cooperative Oceanic Fisheries Investigations Atlas 31.

Navarro-Olache LF, Lavín MF, Alvarez-Sánchez LG, Zirino A. Internal structure of SST features in the central Gulf of California. Deep Sea Res Part II Top Stud Oceanogr. 2004;51(6–9):673–87.

Pedersen M 1985. Puget Sound Pacific whiting, *Merluccius productus*, resource and industry—an overview. Mar Fish Rev 47:35–38

Phillips AJ, Ralston S, Brodeur RD, Auth TD, Emmett RL, Johnson C and Wespestad VG. 2007. Recent pre-recruit Pacific hake (*Merluccius productus*) occurrences in the northern California Current suggest a northward expansion of their spawning area. California Cooperative Oceanic Fisheries Investigations Report 48, 215–229.

Pitcher TJ and Alheit J. What makes a hake? A review of the critical biological features that sustain global hake fisheries. In: Alheit J, Pitcher TJ (eds) Hake. Chapman & Hall Fish and Fisheries Series, vol 15. Springer, Dordrecht

Portela E, Beier E, Barton ED, Castro R, Godínez V, Palacios-Hernández E, et al. Water masses and circulation in the Tropical Pacific off central Mexico and surrounding areas. J Phys Oceanogr [Internet]. 2016 Oct [cited 2016 Dec 23];46(10):3069–81. Available from: http://journals.ametsoc.org/doi/10.1175/JPO-D-16-0068.1

Quinn T, 2010. An environmental and historical overview of the Puget Sound ecosystem, in Shipman H, Dethier MN, Gelfenbaum G, Fresh KL, and Dinicola RS, eds., 2010, Puget Sound Shorelines and the Impacts of Armoring—Proceedings of a State of the Science Workshop, May 2009: U.S. Geological Survey Scientific Investigations Report 2010-5254, p. 11-18

Sánchez-Velasco L, Lavín MF, Peguero-Icaza M, León-Chávez CA, Contreras-Catala F, Marinone SG, et al. Seasonal changes in larval fish assemblages in a semi-enclosed sea (Gulf of California). Cont Shelf Res. 2009;29(14):1697–710.

Santiago-García MW, Marinone SG, Velasco-Fuentes OU. Three-dimensional connectivity in the Gulf of California based on a numerical model. Prog Oceanogr. 2014;123:64–73.

Saunders MW and McFarlane GA. 1997. Observations on the spawning distribution and biology of offshore Pacific hake. CalCOFI Reports, 38: 147–157.

Shipman H. 2008. A geomorphic classification of Puget Sound nearshore landforms: Seattle, Wash., U.S. Army Corps of Engineers, Puget Sound Nearshore Partnership Report No. 2008-01.

Smith PE. 1995. Development of the population biology of Pacific hake, *Merluccius productus*. CalCOFI Rep. 36:144-452.

Smith PE, Horne JK and Schneider DC. 2001. Spatial dynamics of anchovy, sardine and hake pre-recruit stages in the California Current. ICES Journal of Marine Science, 58: 1063–1071.

Waples RS, Pess GR and Beechie T. 2008. Evolutionary history of Pacific salmon in dynamic environments. Evolutionary Applications 1(2), 189-2006.

Ware DM, and Thomson RE. 2005. Bottom up ecosystem trophic dynamics determine fish production in the northeast Pacific. Science, 308: 1280–1284.

Winter DF, Banse K and Anderson GC. 1975. The dynamics of phytoplankton blooms in Puget Sound, a fjord in the Northwestern United States. Marine Biology 29, 139-176
