# Supplementary figures and images for: Role of oceanography in shaping the genetic structure in the North Pacific hake Merluccius productus
Source: PLoS One. 2018 Mar 26;13(3):e0194646. doi: 10.1371/journal.pone.0194646 (PMC5868808; doi:10.1371/journal.pone.0194646)

**Fig A**.


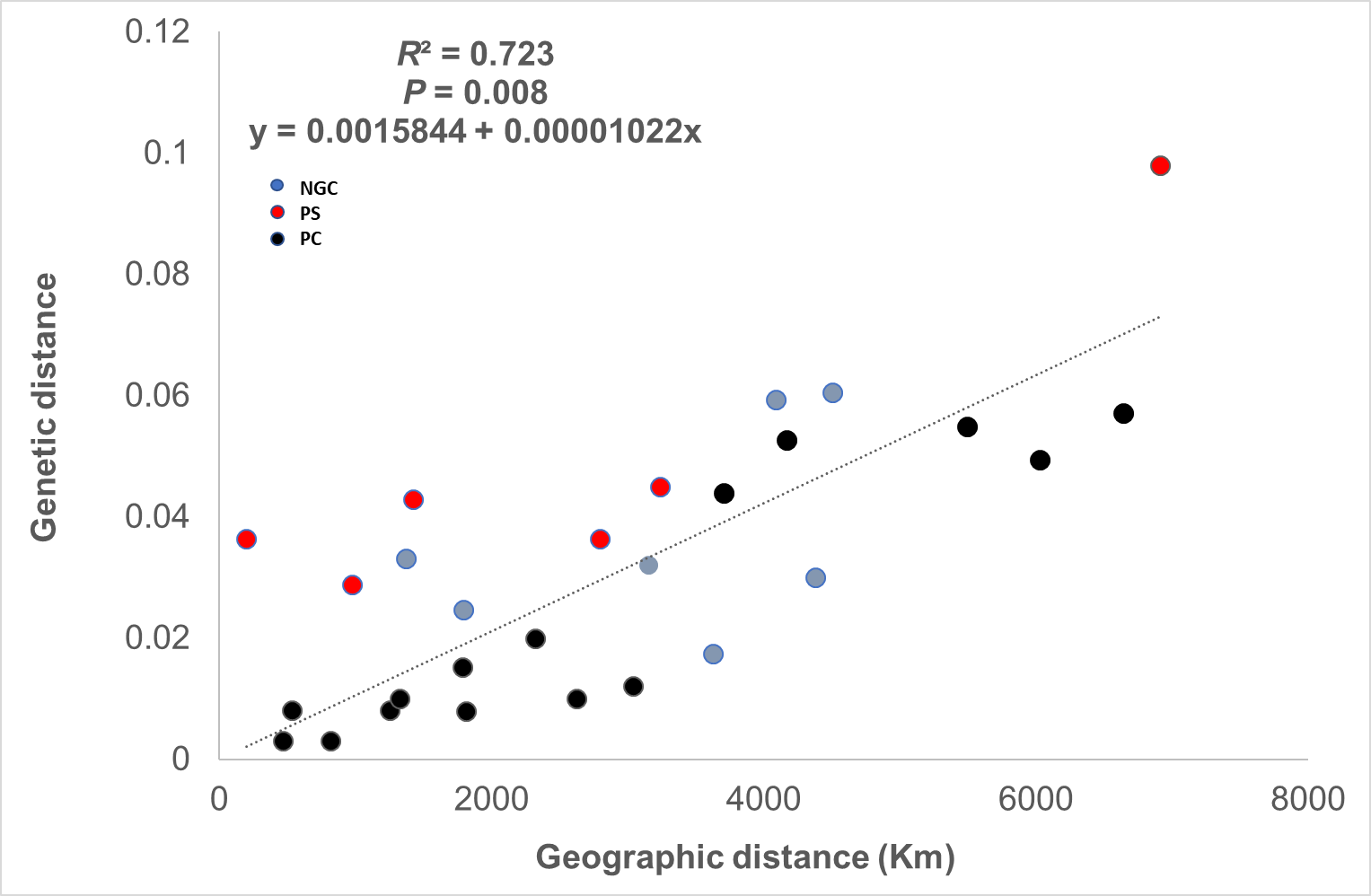


**Fig B.**

**A.**

|  |  |  |  |
| --- | --- | --- | --- |
|  |  | **→** | **←** |
| **PC** | **PS** | 111 (6-332) | 93 (3-287) |
| **PC** | **NGC** | 36 (5- 95) | 54 (5-119) |
| **PS** | **NGC** | 61 (6-209) | 39 (2-103) |
|  |  |  |  |
|  |  |  |  |

**B.**

Supplement: S1 Fig — Fig A. Isolation by distance relationship. Scatter plot of pairwise genetic distance (linearized FST) vs geographic distances of eight microsatellite loci for Merluccius productus showing significant correlation between geographic and genetic distance. Fig B. General patterns of estimates of migration rates between Merluccius productus populations. A. Average estimates of migrants per generation with range in parentheses. Arrows indicates direction of gene flow. B. Depiction of migration rates, with line thickness proportional to migration rate. PC Pacific coast, PS Puget Sound and NGC northern Gulf of California. (DOCX) [file pone.0194646.s004.docx]
